# Effect of Women’s Education, Information and Communication Technologies, and Income on Maternal Mortality: Evidence from BRIICS Countries

**DOI:** 10.3390/healthcare13060602

**Published:** 2025-03-10

**Authors:** Adrian Teodor Moga Rogoz, Gamze Sart, Yilmaz Bayar, Marina Danilina, Marius Dan Gavriletea

**Affiliations:** 1 Department of Maxilo-Facial Surgery and Radiology, University of Medicine and Pharmacy “Iuliu Hatieganu”, 400012Cluj-Napoca, Romania; moga.adrian@umfcluj.ro; 2 Department of Educational Sciences, Hasan Ali Yucel Faculty of Education, İstanbul University-Cerrahpaşa, Istanbul 34500, Turkey; gamze.sart@iuc.edu.tr; 3 Department of Public Finance, Faculty of Economics and Administrative Sciences, Bandirma Onyedi Eylul University, Bandirma-Balikesir 10200, Turkey; 4 Department of Economics, Plekhanov Russian University of Economics (PRUE), 117997 Moscow, Russia; marinadanilina@yandex.ru; 5 Department of Economics, Financial University Under the Government of the Russian Federation, 125167 Moscow, Russia; 6 Department of Business, Business Faculty, Babes-Bolyai University, 400084 Cluj-Napoca, Romania; marius.gavriletea@ubbcluj.ro

**Keywords:** women’s education, ICT, income, maternal mortality, panel econometrics

## Abstract

Maternal mortality has been among one of the most significant global health problems despite noteworthy decreases in maternal mortality during recent decades, and reducing maternal mortality is one of the targets of Sustainable Development Goal 3 (Good Health and Well-being). **Objectives**: This study investigates the effect of women’s education, ICTs (information and communication technologies), and income level on maternal mortality in the BRIICS countries for the 2000–2020 period. **Methods**: Panel causality and regression approaches are employed to analyze the interaction amongst women’s education, ICTs, income level, and maternal mortality. **Results**: The results of the causality test reveal that women’s education, ICTs, and income have a significant influence on the maternal mortality ratio. Specifically, the regression results indicate that women’s education, ICTs, and income have a negative effect on maternal mortality, but women’s education has been identified as one of the most significant factors in reducing maternal mortality ratios. **Conclusions**: Institutional and legal measures to increase women’s education would be useful to globally decrease maternal mortality.

## 1. Introduction

Maternal mortality remains one of the most pressing global health challenges, particularly affecting women from low- and middle-income countries. Despite significant progress in reducing maternal deaths over the past few decades, maternal mortality ratios (MMRs) in many regions remain alarmingly high, reflecting persisting inequalities in access to health services, education, and economic opportunities. According to the World Health Organization (WHO), in 2020, approximately 287,000 women lost their lives due to complications related to pregnancy and childbirth [1], with the vast majority of these deaths happening in low- and lower-middle-income countries. In this context, understanding the socio-economic and systemic determinants of maternal mortality is critical for developing effective strategies to reduce these deaths and to improve maternal health outcomes worldwide.

Previous research consistently shows that women’s education plays a critical role in reducing both maternal mortality [2,3] and early childhood mortality [4,5]. In this context, women’s education can impact maternal health through a variety of perspectives: First, women with higher education levels usually have greater healthy life- and health-related knowledge [6]. Secondly, women with higher education generally have jobs with higher wages and, in turn, they can access better healthcare opportunities [7,8] and, in turn, higher economic gains can cause these women to reduce or to postpone childbearing [9]. However, educated women adopt healthier behaviors during pregnancy [10] if they decide to have a child. Thirdly, increasing the role of women in business may also increase their effect in the family’s decision-making process [11]. As a conclusion, all these educational implications can reduce the maternal mortality ratio through decreases in fertility rates and short birth intervals [12] and decreases in unintended pregnancies [13].

However, the relationship between women’s education and maternal health outcomes is not entirely straightforward. Various socio-economic factors, such as income levels and access to information and communication technologies (ICTs), also significantly influence maternal health [14,15]. ICTs, including mobile phones, the internet, and digital health platforms, can serve as powerful tools for disseminating health information, improving healthcare access, outcomes, and communication between a patient and the healthcare system, alongside international collaboration on healthcare [16,17]. Additionally, economic factors such as household income and national economic conditions affect maternal health by influencing the availability of resources for healthcare, nutrition, and transportation—all of which are vital for reducing maternal mortality.

Given their dynamic economic growth and demographic transformations, the BRIICS countries (Brazil, Russia, India, Indonesia, China, South Africa) provide valuable context for analyzing the relationship between women’s education, ICTs, and income levels on maternal mortality. These rapidly developing nations present considerable differences in their healthcare infrastructure, education systems, income levels, and ICT availability, making them a valuable framework for exploring how different socio-economic factors interact and influence maternal health outcomes.

The present study investigates the effects of women’s education, ICTs, and income on maternal mortality in the BRIICS countries over the period from 2000 to 2020. By employing both causality and regression methods, this research provides empirical evidence of the complex relationships between these variables. The results of our causality analysis reveal that women’s education, ICTs, and income have a significant influence on maternal mortality, with education being a key determinant of lower maternal death rates. Furthermore, the results of the regression analysis also confirm that while all three factors—women’s education, ICTs, and income—exert a negative effect on maternal mortality, women’s education emerges as the most significant factor in driving decreases in maternal deaths.

The structure of this paper follows the following steps: The first section provides a detailed review of the literature on the relationship between women’s education, ICTs, income, and maternal mortality, highlighting the key findings and the research gaps. Next, the research methodology, including the causality and regression approaches used to analyze the data, is discussed. Following this, the study outlines the key findings, with a particular focus on the importance of enhancing women’s education and access to ICTs to improve maternal health outcomes in the BRIICS countries.

Finally, the article concludes by offering recommendations for future research and policy measures to reduce maternal mortality in these regions.

## 2. Literature Review

Maternal mortality has become a vital problem to be addressed, especially for developing and emerging economies. Therefore, unlocking drivers of maternal mortality is crucial for appropriate policy-making. In this study, the causal relationship amongst women’s education, ICTs, income, and maternal mortality in BRIICS countries is investigated. In this context, a few empirical research papers, including Weitzman [2], Mensch et al. [4], Wu [6], McAlister and Baskett [18], Karlsen et al. [19], Ferdos and Islam [20], and Asare [21], exploring the association between women’s education and maternal mortality have uncovered a negative influence of women’s education on maternal mortality. Furthermore, Barman et al. [22] and Amwonya et al. [23] found that women’s education had a positive effect on the utilization of maternal healthcare.

McAlister and Baskett [18] examined the association between women’s education and maternal mortality in 148 countries by means of regression and proved that educational indicators about women were disclosed to be moderate determinants of maternal mortality. On the other hand, Karlsen et al. [19] investigated the interplay between women’s education and maternal mortality among women giving birth in healthcare institutions by utilizing data from the WHO Global Survey on Maternal and Perinatal Health and discovered that women having lower education levels was related to higher levels of maternal mortality.

Weitzman [2] analyzed the causal relation between women’s education and maternal health in Peru across the 2003–2009 period by means of regression and found that increases in women’s schooling years reduced maternal mortality. On the other hand, Mensch et al. [4] analyzed 16 empirical studies related to the nexus between education and maternal and child health and unveiled that women’s education had a significant effect on maternal health.

Ferdos and Islam [20] explored the effect of women’s education on maternal health in Bangladesh by reviewing 25 studies and revealed that improvements in women’s education decreased maternal mortality. Wu [6] also investigated the effect of women’s education on child mortality in Bangladesh by employing data from the 2007, 2011, and 2014 Bangladesh Demographic and Health Surveys through a regression approach and showed that progress in women’s education reduced child mortality. Asare [21] examined the effect of women’s education on maternal deaths in the eastern part of Ghana between 2011 and 2016 by means of a retrospective approach and uncovered that maternal deaths were relatively lower among women with senior high school and tertiary school education.

Barman et al. [22] examined the impact of women’s education on the use of maternal healthcare services in India and unveiled a positive impact of women’s education on the use of maternal health services. Amwonya et al. [23] also explored the effect of women’s education on the utilization of maternal healthcare in Uganda by using data from questionnaires conducted between 2006 and 2011 through regression and found a positive effect of women’s education on the use of maternal healthcare.

Based on theoretical considerations and associated empirical results, our first research hypothesis was as follows:

**H1.**  *Women’s education has a significant effect on maternal mortality*.

ICT penetration has remarkably increased in recent years, but the effect of ICT indicators on maternal health has been examined by few researchers. In this context, Mlambo et al. [15] analyzed the effect of ICTs on maternal mortality in the SADC (Southern African Development Community) countries across the 2000–2018 period by means of dynamic regression, pooled mean group, and mean group estimators and unveiled that improvements in ICTs represented by mobile cellular telephone subscriptions decreased maternal mortality. Shao et al. [24] also explored the effect of ICT indicators on health indicators in 141 countries over the period of 2012–2016 by means of regression analysis and found that improvements in the ICT environment decreased the maternal mortality ratio.

Vaidean and Achim [25] studied the effect of ICTs proxied by internet usage and mobile cellular subscriptions on health indicators in 158 countries for the 2005–2018 period by means of regression and showed that increases in ICT indicators decreased maternal mortality. Lastly, De and Pradhan [26] reviewed 25 studies on the nexus between mobile technology and the use of maternal healthcare in low- and middle-income countries and uncovered that mobile technology had a positive effect on the use of maternal healthcare in these countries, but some studies also pointed out problems related to technology use and misuse, poor–rich discrimination, and inequality in phone ownership.

Based on these theoretical considerations and associated empirical results, the second hypothesis of this research is as follows:

**H2.**  *ICT has a significant effect on maternal mortality.*

Income level and maternal mortality are closely interrelated. Therefore, some researchers have investigated the influence of maternal mortality on economic growth and development. In this context, Kirigia et al. [27] and Nnadi et al. [28] unveiled a negative effect of maternal mortality on economic growth and development. On the other hand, some researchers have focused on the influence of income on maternal mortality, and Jeong et al. [14] and Aziz et al. [29] showed that increases in income level decreased maternal mortality. However, Islam et al. [30] uncovered an insignificant causality between maternal mortality and economic growth.

Jeong et al. [14] explored the interaction between income level and maternal mortality in South Korea between 2003 and 2013 using the log-binomial regression and showed that women with lower income levels usually had a higher risk of maternal mortality. On the other hand, Aziz et al. [29] explored determinants of maternal mortality in South Asian countries across the 2000–2017 period by means of FMOLS (fully modified ordinary least squares) and DOLS (dynamic ordinary least squares) estimators and showed that increases in economic growth decreased maternal mortality. Last, Islam et al. [30] investigated the long-term relationship between economic growth and health indicators in Saudi Arabia for the 1990–2019 by means of Johansen’s cointegration test and revealed an insignificant causality between maternal mortality rate and economic growth.

Based on these theoretical considerations and associated empirical results, the third hypothesis of this research is as follows:

**H3.**  *Income has a significant effect on maternal mortality.*

## 3. Data and Methodology

This study investigates the effect of women’s education, ICTs, and income on maternal mortality in the BRIICS countries across the period of 2000–2021 by means of causality and regression approaches. The variables utilized in the experimental part of this article are introduced in Table 1. Maternal mortality is proxied by the maternal mortality ratio, which is calculated as the number of maternal deaths per 100,000 live births during a given time period and acquired from the World Health Organization [31]. On the other hand, women’s education is represented by means of women’s schooling years and obtained from the UNDP (United Nations Development Programme) [32]. In the related literature, researchers have used internet usage rate by the population, mobile–cellular telephones, and fixed broadband subscriptions as proxies of ICTs [15,24,25]. However, this study uses the ICT index of UNCTADSTAT (United Nations Trade and Development (UNCTAD) Statistics) [33] for information and communication technologies (ICTs) because this index is calculated as a combination of fixed line and mobile phone users, internet accessibility, and server security in a similar way to studies by Saba et al. [34] and Zelenkov and Lashkevich [35]. The ICT index is acquired from UNCTADSTAT (2024). Finally, income is represented by GDP per capita (constant 2015 USD) and obtained from the World Bank [36].

The empirical analysis is based on the BRIICS countries, and the empirical analysis is limited to the period of 2000–2020, as the ICT index, calculated by UNCTADSTAT, is available only up to 2020, and maternal mortality data extend through 2020. Econometric analyses were conducted using EViews 12.0 and Stata 15.0.

The mean values of maternal mortality, the mean schooling years for women, the ICT index, and income in all BRIICS countries over the 2000–2020 period are, respectively, 123.243 maternal deaths per 100,000 live births, 7.595 years, 35.606, and USD 5353.903, as introduced in Table 2. However, there are significant differences in maternal mortality ratios, real GDP per capita, and the ICT index among the BRIICS countries, but only the mean schooling years for women variable displays a moderate variation among the BRIICS countries.

In the experimental part of this paper, CSD (cross-sectional dependency) and heterogeneity tests were firstly conducted. Then, unit root, causality test, and regression analysis were performed to examine the relationship among maternal mortality, women’s education, ICT index, and income. The Emirmahmutoglu and Kose [37] causality test is a modified panel version of Toda-Yamamoto’s [38] causality test under the presence of CSD and heterogeneity. The estimated VAR (vector autoregression) model for each cross-section is as follows:

(1) $$y_{it}=\delta_{i}+A_{1i}y_{i\left(t-1\right)+\ldots +}A_{pi}y_{i\left(t-pi\right)+\ldots +}A_{(p+d)i}y_{i(t-pi-di)}+u_{i,t}\;$$

In Equation (1), $y_{it}$ is an endogenous variable, $\delta_{i}$ is a fixed-effects vector consisting of p dimensions, p_i_ is the optimal lag length, and d_i_ is the highest integration level of the variables of MATERNAL, MYS, ICT, and INCOME [35]. In addition, the causality test enables the lag length to become different for each cross-section and in turn reduces the long-term information loss due to modeling the series with level values [37].

Lastly, regression analysis was also performed to unravel the effect of women’s education, the ICT index, and income on maternal mortality. In this context, a fixed-effects model was utilized regarding the results of the Breusch and Pagan [39] LM test, the Chow [40] F test, and the Hausman [41] test, but the problems of heteroscedasticity and serial correlation directed us to make a choice between the Driscoll-Kraay estimator, the Parks–Kmenta estimator, and the Beck–Katz estimator, resulting in more reliable results in the case of heteroscedasticity and serial correlation. The Driscoll–Kraay estimator culminates in relatively more reliable results in the case that the cross-section size is greater than the time range, but the Parks–Kmenta estimator results in more reliable results in the case that the time range for the dataset is greater than the cross-sectional size of the dataset [42]. In this research article, the Parks–Kmenta estimator is chosen because the time dimension of our dataset was greater than its cross-section dimension.

## 4. Results of Econometric Analysis and Discussion

In the experimental part of this research, the availability of collinearity in the model was first investigated by means of correlation and VIF analyses. In this context, the correlation matrix amongst the explanatory variables and variance inflation factor (VIF) scores were calculated and are exhibited in Table 3. A VIF value which is greater than 5 or 10 points to a collinearity problem [43]. Therefore, the low correlation and VIF values in Table 3 demonstrate the non-availability of the collinearity problem in this model.

The presence of CSD and heterogeneity was analyzed by means of the LM (Lagrange multiplier) and delta tilde tests. Therefore, LM_adj._, LM CD, and LM tests were applied, and the null hypothesis of independence was rejected because the probability values of these, as presented in Table 4, were lower that 5%. Then, delta tilde tests were applied, and the null hypothesis of homogeneity was rejected. To conclude, our results provide evidence of CSD and heterogeneity among MATERNAL, MYS, ICT, and INCOME.

The utilization of ordinary least squares or other similar approaches for non-stationary time series may result in spurious outcomes, and the estimated regression results may reveal a significant relationship between two variables which are uncorrelated [44]. Therefore, performing a stationarity analysis is crucial prior to regression and causality analyses. Given the presence of CSD and heterogeneity, it is essential to test for the presence of the unit root in the series of MATERNAL, MYS, ICT, and INCOME, and this is also required for causality and regression. Consequently, the panel CIPS unit root test devised by Pesaran [45] was employed, with the results presented in Table 5. The findings indicate that MATERNAL, MYS, ICT, and INCOME are I(1), suggesting that these variables are non-stationary at this level but are stationary at the first difference.

The causal links among women’s education, ICTs, income, and maternal mortality are examined using the causality test created by Emirmahmutoglu and Kose [37] given the presence of CSD and heterogeneity among the variables under consideration, and the results are reported in Table 6, Table 7 and Table 8. The causality analysis between MYS and MATERNAL, as presented in Table 5, reveals a unidirectional causal relationship running from women’s years of schooling to maternal mortality at the panel level, in China and South Africa. In other words, the results demonstrate that women’s education has a significant influence on the maternal mortality ratio.

The causality analysis between ICT and maternal mortality, as presented in Table 7, reveals a unidirectional causal relationship running from the ICT index to maternal mortality at the panel level, in Indonesia and South Africa.

The causality analysis between INCOME and maternal mortality, as presented in Table 8, reveals a unidirectional causal relationship running from income to maternal mortality at the panel level. In other words, income has a significant influence on the maternal mortality ratio.

The impact of women’s education, the ICT index, and income on the maternal mortality ratio was also examined by means of the Parks–Kmenta estimator, a more robust estimator for addressing the presence of autocorrelation and heteroscedasticity. The estimated coefficients are presented in Table 9. The results indicate that improvements in the mean schooling years for women, the ICT index, and income all led to a decrease in the maternal mortality ratio. Among these, the mean years of schooling variable had the most significant effect on maternal mortality, followed by ICT, which had a moderate influence. In contrast, income, measured by real GDP per capita, exerts a relatively weaker effect on maternal mortality. The adjusted R-square value of 0.721 indicates that the model consisting of education, income, and ICTs had significant predictive power over maternal mortality. Furthermore, the same model was estimated by the Driscoll–Kraay estimator for the robustness of the results, and the coefficients revealed that increases in the mean schooling years, ICTs, and income led to a decrease in maternal mortality, but the effect of mean schooling years on maternal mortality was relatively lower, while the effect of ICT and income on maternal mortality was relatively greater when compared with the coefficients determined by the Parks–Kmenta estimator. However, the similar direction of effect and adjusted R-square value support the results obtained by the Parks–Kmenta estimator.

The results of the regression analysis are given to support the findings of the causality analysis. In this context, women’s education is a vital factor for decreases in maternal mortality because improvements in education can decrease maternal mortality through enhancing women’s awareness about healthy life, access to health-related knowledge, and healthcare opportunities. As a consequence, healthier behaviors during pregnancy, decreases in short birth intervals, fertility rates, and unintended pregnancies contribute to decreases in maternal mortality. Furthermore, Weitzman [2], Wu [6], McAlister and Baskett [18], and Karlsen et al. [19] also identified education as a significant determinant of improvements in maternal mortality. However, Barman et al. [22] and Amwonya et al. [23] revealed a positive influence of women’s education on the use of maternal health services. Our results from the causality and regression analyses demonstrate that education is the most dominant determinant of decreases in maternal mortality and are consistent with the empirical results in the related literature. Therefore, our results support the validity of the first hypothesis of this study based on the related theoretical and empirical literature.

ICT penetration has remarkably increased over the world, has served as a powerful tool for disseminating health information, and has been useful in improving healthcare access, outcomes, and communication between patient and healthcare systems, alongside improving international collaboration on healthcare. In addition, the related limited empirical studies by Mlambo et al. [15], Shao et al. [24], and Vaidean and Achim [25] also obtained results supporting these theoretical considerations for panels including countries from different parts of the world. However, the literature review conducted by De and Pradhan [26] drew attention to problems related to technology use and misuse, poor–rich discrimination, and inequality in phone ownership. Our results from the causality and regression analyses also unveil ICT as a significant determinant of decreases in maternal mortality and are consistent with the empirical results in the related literature. Therefore, our results support the validity of the second hypothesis of this study based on the related theoretical and empirical literature.

Lastly, income is expected to decrease maternal mortality through improvements in nutrition and access to better healthcare services. In a similar way, Jeong et al. [14] and Aziz et al. [29], respectively, discovered that increases in income level decreased maternal mortality in South Korea and South Asian countries. On the other hand, Kirigia et al. [27] and Nnadi et al. [28] found a negative effect of maternal mortality on economic growth. Our regression results are compatible to the results of Jeong et al. [14] and Aziz et al. [29]. Lastly, Islam et al. [30] disclosed an insignificant causal nexus between economic growth and maternal mortality, differently from our significant causality between income level and maternal mortality. In conclusion, our results support the validity of the third hypothesis of this study based on the related theoretical and empirical literature.

## 5. Conclusions, Limitations, and Policy Recommendations

Maternal mortality is still one of the most serious global health problems that need to be addressed, despite the remarkable decrease in maternal mortality. Therefore, this study investigates the effect of women’s education, ICTs, and income on maternal mortality in the BRIICS countries across the 2000–2020 period by means of regression and causality approaches. The results of the causality test point out the significant influence of women’s education, ICTs, and income on the maternal mortality ratio. On the other hand, the results of the regression analysis conducted by means of the Parks–Kmenta and Driscoll–Kraay estimators verify the findings of the causality test and show that improvements in women’s education, ICT, and income decrease maternal mortality. In addition, women’s education has been identified as one of the most significant factors in reducing maternal mortality rates when compared to ICT and income. Furthermore, the adjusted R-square value of 0.721 indicates that education, income, and ICT have significant predictive power for maternal mortality.

The limitations of this study are as follows:

The study sample includes only BRIICS countries.

The study period is limited to the 2000–2020 period due to the availability of maternal mortality and ICT data.

The main focus of this study was analyzing the interplay among education, ICTs, income, and maternal mortality. Therefore, the effect of global, regional, and national economic crises and conflicts, such as the 2008 global financial crisis, the Eurozone sovereign debt crisis, the Russian financial crisis, the 2014 Brazilian economic crisis, the 11 September attacks, and other socio-economic parameters, on maternal mortality via income, access to food, and healthcare services was not considered.

Based on our results and the related literature, governments must address the maternal health crisis and implement policy measures aimed at reducing maternal mortality in BRIICS countries.

Invest in women’s education:

-Since our research indicates that education is a significant determinant in reducing maternal mortality, the expansion of educational opportunities for women at all levels should be prioritized. Enhancing access to secondary and higher education for women, particularly in rural and underserved areas, can improve maternal health outcomes by increasing health literacy, promoting family planning, and encouraging healthier lifestyle choices.

-Community-based educational programs that target adult women should be introduced, especially those with low levels of formal education, to ensure they gain vital knowledge on maternal health and safety during pregnancy and childbirth.

Enhance ICT infrastructure:

-Investments in ICT infrastructure should be increased to improve access to maternal health services and information. Digital platforms can provide remote consultations, education, and updates on maternal health risks. Also, mobile health apps can be used to provide pregnant women with health guidance [46], track vital signs, and send reminders.

-Governments should collaborate with private entities and international organizations to develop telemedicine and mobile health solutions that can help reduce maternal mortality, especially in regions where health professionals are scarce.

Increase income and economic empowerment for women:

-Policies should be implemented that facilitate access to formal employment, ensure equal remuneration for equal work, and support microfinance programs that focus on small business development. Economic empowerment allows women to make better health choices and afford maternal healthcare services.

-Support should be provided for women in low-income households by providing financial assistance and health insurance programs that cover maternity and postnatal care costs.

Strengthen healthcare systems:

-Health facilities should be improved, providing adequate training for healthcare workers.

-It is vital to ensure that births are attended by skilled health personnel.

Strengthen maternal health policies and awareness campaigns:

-National awareness campaigns should be launched to educate the public on maternal health, focusing on the importance of education, timely healthcare, and safe childbirth practices.

Governments should implement policies and regulations that promote gender equality and protect women’s rights in all aspects of life, including access to education, employment, and healthcare services.

In conclusion, by combining the strengths of education, ICTs, and economic empowerment, BRIICS countries have the potential to make substantial progress in reducing maternal mortality rates. This integrated approach can lead to improved maternal health outcomes, contributing to the broader goal of sustainable development and economic stability in the region.

## Figures and Tables

**Table 1:** Definition of the variables.

Variables	Explanation	Source
MATERNAL	Maternal deaths per 100,000 live births	World Health Organization [31]
MYS	Mean schooling years for women (years)	UNDP [32]
ICT	Index of information and communication technologies	UNCTADSTAT [33]
INCOME	Real GDP per capita (constant 2015 USD)	World Bank [36]

**Table 2:** Summary statistics (BRIICS countries; 2000–2020).

Variables	Mean Value	Std. Dev.	Min	Max
MATERNAL	123.243	95.672	7.453	384.448
MYS	7.595	2.736	3.041	12.470
ICT	35.606	15.652	9.283	69.59
INCOME	5353.903	2941.712	756.704	10,358.17

**Table 3:** Correlation matrix (BRIICS countries; 2000–2020).

	MYS	ICT	INCOME	VIF
MYS	1	0.325 **	0.304 **	2.574
ICT		1	0.216 **	3.009
INCOME			1	1.956

** indicates statistical significance at the 5%.

**Table 4:** Outcomes of CSD and slope homogeneity tests (BRIICS countries; 2000–2020).

CD Tests		Slope Homogeneity Tests	
Test	Test Statistic	Test	Test Statistic
LM_adj._	4.981 ***	Delta	10.037***
LM CD	2.562 **	Bias-Adj. Delta	11.499***
LM	29.35 ***		

*** and ** indicate statistical significance at the 1% and 5% levels, respectively.

**Table 5:** CIPS test outcomes (BRIICS countries; 2000–2020).

Variables	Level		First Differenced Values	
	Constant	Constant + Trend	Constant	Constant + Trend
MATERNAL	−0.601	0.442	−4.157 ***	−3.094 ***
MYS	−0.052	−0.617	−3.504 ***	−1.709 **
ICT	0.844	1.611	−4.883 ***	−3.286 ***
INCOME	−0.014	2.471	−3.411 **	−4.056 ***

*** and ** indicate statistical significance at the 1% and 5% levels, respectively.

**Table 6:** Outcomes of causal test between MYS and MATERNAL (BRIICS countries; 2000–2020).

Countries	Lag	MYS ↛ MATERNAL		MATERNAL↛ MYS	
		Test Statistic	*p* Value	Test Statistic	*p* Value
Brazil	1	1.154	0.283	0.661	0.416
China	3	12.787	0.005	3.086	0.379
India	1	0.970	0.325	0.563	0.453
Indonesia	1	1.413	0.235	0.007	0.934
Russia	3	3.815	0.282	4.203	0.240
South Africa	2	4.614	0.045	1.695	0.429
Panel		25.371	0.013	9.962	0.619

Note: optimal lags are determined by Akaike information criterion. ↛: X is not Granger cause of Y.

**Table 7:** Outcomes of causal test between ICT and MATERNAL (BRIICS countries; 2000–2020).

Countries	Lag	ICT ↛ MATERNAL		MATERNAL↛ ICT	
		Test Statistic	*p* Value	Test Statistic	*p* Value
Brazil	2	2.487	0.288	0.460	0.795
China	1	0.023	0.985	2.003	0.157
India	1	0.103	0.748	0.060	0.807
Indonesia	2	11.737	0.003	0.983	0.612
Russia	3	4.284	0.232	4.100	0.251
South Africa	2	14.637	0.001	0.290	0.865
Panel		32.391	0.001	8.631	0.734

Note: optimal lags are determined by Akaike information criterion. ↛: X is not Granger cause of Y.

**Table 8:** Outcomes of causal test between INCOME and MATERNAL (BRIICS countries; 2000–2020).

Countries	Lag	INCOME ↛ MATERNAL		MATERNAL↛ INCOME	
		Test Statistic	*p* Value	Test Statistic	*p* Value
Brazil	2	0.311	0.856	2.273	0.321
China	3	2.984	0.394	2.402	0.493
India	3	1.551	0.671	2.951	0.399
Indonesia	1	0.097	0.756	1.016	0.313
Russia	3	1.496	0.683	2.253	0.522
South Africa	1	0.609	0.435	0.009	0.925
Panel		25.958	0.018	9.301	0.677

Note: Optimal lags are determined by Akaike information criterion. ↛: X is not Granger cause of Y.

**Table 9:** Regression estimations (BRIICS countries; 2000–2020).

Independent Variables	Parks–Kmenta Estimator	Driscoll–Kraay Estimator
	Coefficients	Coefficients
MYS	−3.752 ***	−1.358 ***
ICT	−0.806 ***	−0.831 ***
INCOME	−0.1274 ***	−0.317 ***
C	206.380 ***	7.463 ***
Wald statistics	95.04 ***	
R-square	0.723	0.744
Adjusted R-square	0.721	0.742

*** indicates statistical significance at 1%.

## Data Availability

The data of this research were acquired from the databases of UNCTADSTAT, UNDP, World Bank, and the World Health Organization.

## Abbreviations

The following abbreviations are used in this manuscript:

BRIICS	Brazil, Russia, India, Indonesia, China, South Africa
CSD	Cross-sectional dependency
DOLS	Dynamic ordinary least squares
FMOLS	Fully modified ordinary least squares
ICT	Information and communication technologies
SADC	Southern African Development Community
UNCTADSTAT	United Nations Trade and Development Statistics
UNDP	United Nations Development Programme
WHO	World Health Organization
VAR	Vector autoregression
VIF	Variance inflation factor
